# Pivot Teeth

**Published:** 1862-08

**Authors:** W. H. Shadoan


### PIVOT TEETH.

BY W. H. SHADOAN.

Pivoting teeth on fangs is objectionable in most cases, though it may be done sometimes with great satisfaction to both patient and dentist.

It is the fewest number of cases that present themselves to us for such operations that are sufficiently sound to fit pivot teeth to, to make a good job. The dentist that refuses all such operations is in as good "fix" as one who refuses to fill teeth with anything else than gold. One reason for not pivoting teeth is the utter impossibility of making a complete fit of the crown to the root. Consequently, small foreign substances will naturally find their way into the interstices, and as a legitimate consequence, cause the root to decay more rapidly than before the artificial crown was attached.

It is regarded by all good dentists that the old style clasp work is objectionable, from the fact that they not only wear away the enamel of the teeth so clasped, but that they hold the small particles of food and other extraneous substances between the clasp and tooth, which is all the while saturated with the saliva, which in all cases is more or less impregnated with some form or other of acid (cyptic or nitric), that corrodes the tooth, and the loss of the tooth and work is the natural result. This class of dentists rarely ever resort to pivot work. In an article in the April number of the Register, Dr. J. Richardson proposes to substitute rubber for wood in pivoting teeth, which I think an excellent idea. For a description of his process, you are referred to the April No. of Register, p. 163.

This plan of inserting pivot teeth so completely excludes all particles of food, etc., that the tooth will necessarily last much longer, and be of far more service than the old way of using wood pivots. One great advantage is in having a perfect fit, an uniform bearing on the fang, which adds greatly to its strength. It is true enough that this kind of work is much more difficult to make, and takes more labor, but when done, is of far more value to the patient, who will remunerate his dentist for the time and labor of doing the work. If a pivot tooth inserted the old way is worth two dollars, one inserted on Dr. Richardson's plan will be worth at least four dollars. It will cost the dentist that much more in time, labor and materials, and as a matter of justice, he ought to be paid for it. As to the duration of pivot work, it is altogether owing to the condition of the root and the manner in which the work is done. If the root be solid, well set, of firm texture, and the crown properly attached, we expect it to be a good job, and will be likely to last a considerable time under favorable circumstances. If the reverse, then the work will be worthless. I extracted a tooth a few days ago, that had a pivot crown to it, that had been there fourteen years, and had never been removed in all that time. I tried first to pull off the crown with my fingers, but found it too tight. I then extracted the tooth, and found it very tight. There was a deposit of salivary calculi between the crown and fang that made the fit a perfect one, and kept all secretions from the fang, and the result was, the fang remained sound. I met a lady in Indiana in 1857, who had worn one pivot tooth twenty-one years, and had only had it reset twice; one time was at the time I saw her. A few out of a great many will last ten, fifteen, and sometimes twenty years, but the majority will only last from three to five years. If a person has only one or two teeth decayed, and no signs of ulceration at the roots, with teeth of hard variety, and they not adjoining each other, they may be pivoted, otherwise I prefer suction plate for substitutes.

